# Expression of the DELLA repressor GAI and its regulators SPY and SEC are impacted by disruption of chromatin modifiers

**DOI:** 10.17912/micropub.biology.000175

**Published:** 2019-10-17

**Authors:** Natalie Trachtman, Patrick Sockler, Hanna Caiola, Elizabeth R McCain, Amy T Hark

**Affiliations:** 1 Biology Department, Muhlenberg College, Allentown, PA 18104, USA; 2 Department of Cell Biology, Rowan University School of Osteopathic Medicine, Stratford, NJ 08084, USA

**Figure 1:**
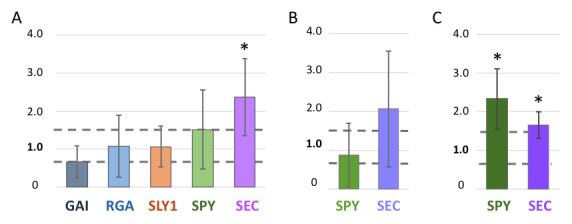
Expression of key DELLA repressors and their regulators in *gcn5* and *ada2b* mutant backgrounds. For each experiment the expression of the target gene in a wildtype background was set to 1 and the expression in the mutant background is calculated relative to that, with upregulation signified by numbers greater than 1 and downregulation by numbers less than 1. Gray dashed lines indicate the position of a 1.5-fold change. Bars show the average expression from 4-6 experiments; error bars show calculated standard deviation. Asterisks indicate a statistically significant difference as measured by Student’s t-test (p<0.05). A. Quantitative RT-PCR (qRT-PCR) results for GAI, RGA, SLY1, SPY, and SEC in *gcn5-6* mutants. B, C. qRT-PCR analysis of SPY and SEC in *gcn5-1* (B) and *ada2b-1* (C) backgrounds.

## Description

Since trichome number is altered with disruption of GCN5 or ADA2b (Kotak *et al.* 2019; Wang *et al.* 2019), we sought to determine whether these chromatin factors might impact the expression of known regulators of trichome initiation, including two members of the DELLA family of repressor proteins, GA-Insensitive (GAI) and REPRESSOR of *ga1-3* (RGA). As their names suggest, these proteins are sensitive to gibberellin (GA) signaling (Silverstone et al. 2001). GA complexed with its receptor can bind to DELLA proteins and promote their interaction with a component of an E3 ubiquitin ligase, SLEEPY1 (SLY1), leading to subsequent proteasome-media degradation (Dill *et al.* 2004). In addition to SLY1, we also assessed the expression of SPINDLY (SPY) and SECRET AGENT (SEC), both known to covalently modify RGA and impact its functionality in a positive and negative manner respectively (Zentella *et al.* 2016, 2017).

In mature rosette leaves of *gcn5-6* plants (harboring a T-DNA insertion that disrupts the catalytic domain of GCN5; Kotak *et al.* 2018), we detect a trend of decreased expression of GAI, while no consistent change in expression is seen for RGA. Among the factors that influence the activity of the DELLA repressors, SLY1 showed no change in expression. On average, there was a 1.5X increase in SPY expression in *gcn5-6* mutants while SEC expression was more than doubled (Fig. 1A). Expression analysis comparing the Columbia (wildtype) and *gcn5-6* background included five biological replicates, except for SLY1, where n=4.

A microarray experiment conducted by Vlachonasios et al. 2003 provides some additional data on these genes’ expression in *ADA2b* and *GCN5* disruption mutants. Specifically, in *ada2b-1* and *gcn5-1* plants (Vlachonasios *et al.* 2003), GAI expression is slightly decreased (less than three-fold) while RGA expression is unchanged, paralleling what we report here with *gcn5-6*. SLY1 expression was also slightly decreased in *ada2b-1* and *gcn5-1* plants. Since SPY and SEC were not included in the microarray experiment, we assayed their expression via qRT-PCR. *SPY* expression is unchanged in *gcn5-1*. This is consistent with the lack of a significant change in *gcn5-6* plants. In *ada2b-1* plants, SPY expression is increased more than 2-fold. *SEC* expression is increased in both *gcn5-1* and *ada2b-1* in comparison to Ws (wildtype) controls. These experiments included 4-6 biological replicates.

## Reagents

*Arabidopsis thaliana* plants were germinated in soil and grown at 22°C under continuous light conditions (140 µmoles/m^2^/sec). Plants were watered once a week with Hoagland’s solution and a second time weekly (or as needed) with deionized water. Plants were genotyped via PCR as described previously (Kotak *et al.* 2018) and rosette leaves were harvested at 4-5 weeks of age and flash frozen in liquid nitrogen prior to preparation of RNA (Qiagen Plant RNeasy kit). Between 0.5-1 µg of RNA was used in each cDNA synthesis reaction (Maxima H Minus cDNA Synthesis Master Mix with dsDNAse, Thermo Scientific). Quantitative PCR was performed using an Applied Biosystems StepOne Plus instrument with the FAST SYBR Green Master Mix. Primer sequences used to amplify each target cDNA as well as two “housekeeping” genes, At4g26410 (used in GAI and RGA trials) and At1g13440 (used in SLY1, SPY, and SEC trials) are listed in the Table below. Comparative Ct analysis that includes normalization of wildtype (Ws-2 or Col-0) and mutant samples based on expression of the housekeeping gene was conducted using the Applied Biosystems instrument software. Melting temperature analysis confirmed the presence of a single amplicon in each reaction. Each data point within an experiment was performed in triplicate, with an allowable standard deviation of Ct values < 5%. Average Ct values for the housekeeping gene for wildtype and mutant samples needed to be within three cycles of one another for data to be included in this analysis.

**Table:** 

Gene name	Locus identifier	Forward Primer	Reverse Primer
GAI	AT1G14920	CTGTGGTTGAGCAGGAATCG	AACCTCCGACATGACCTTGT
RGA	AT2G01570	GAAAGTTCTCGGCGTTGTGA	CTTGCTCAACCACCGTGAAA
SLY1	AT4G24210	TGCTCACAACGAGACAAACA	TGCTCACGCAAGATGACATA
SPY	AT3G11540	TTGAGGCTCACAGAGACTGG	GGTGATAGGTCGCTCTGGAT
SEC	AT3G04240	TCTGCTATGTCATCTGATGCTATCG	GGAAGCCCATATATGAAACCTGGAT
RHIP1	AT4G26410	GAGCTGAAGTGGCTTCCATGAC	GGTCCGACATACCCATGATCC
GAPC2	AT1G13440	TTGGTGACAACAGGTCAAGCA	AAACTTGTCGCTCAATGCAATC
